# The B Cell Response and Formation of Allergenic and Anti-Allergenic Antibodies in Food Allergy

**DOI:** 10.3390/biology12121501

**Published:** 2023-12-07

**Authors:** Christopher C. Udoye, Marc Ehlers, Rudolf A. Manz

**Affiliations:** 1 Institute for Systemic Inflammation Research, University of Lübeck, 23562 Lübeck, Germany; 2 Laboratories of Immunology and Antibody Glycan Analysis, Institute for Nutritional Medicine, University of Lübeck and University Medical Center Schleswig-Holstein, 23538 Lübeck, Germany

**Keywords:** food allergy, IgA, IgG, IgE, anaphylaxis, B cell

## Abstract

**Simple Summary:**

It has long been known that antibodies of a certain class, called IgE, are the main cause of allergic reactions to food. Other antibody types such as IgA and IgG can suppress the allergic reaction. Even IgE, which binds the allergen only weakly (i.e., with low affinity), seems to have a protective effect. This review discusses the role of distinct antibody types in food allergy, and the underlying mechanisms of action. These findings might be important to understand the development and course of food allergies, and could help to improve diagnostics and therapy.

**Abstract:**

Food allergies are a growing public health concern worldwide, especially in children and young adults. Allergen-specific IgE plays a central role in the pathogenesis of food allergies, but their titers poorly correlate with allergy development. Host immune systems yield allergen-specific immunoglobulin (Ig)A, IgE and IgG subclasses with low or high affinities and differential Fc *N*-glycosylation patterns that can affect the allergic reaction to food in multiple ways. High-affinity IgE is required to induce strong mast cell activation eventually leading to allergic anaphylaxis, while low-affinity IgE can even inhibit the development of clinically relevant allergic symptoms. IgA and IgG antibodies can inhibit IgE-mediated mast cell activation through various mechanisms, thereby protecting IgE-positive individuals from allergy development. The production of IgE and IgG with differential allergenic potential seems to be affected by the signaling strength of individual B cell receptors, and by cytokines from T cells. This review provides an overview of the diversity of the B cell response and the diverse roles of antibodies in food allergy.

## 1. Introduction

Food allergies are becoming an increasingly global health concern, especially in urbanized areas, with the prevalence of food allergies rising by about 1.7% between 1997 and 2011 [1]. Allergic reactions to a food antigen are thought to be mainly driven by IgE-mediated activation of mast cells and basophils. They account for most allergies, resulting in symptoms ranging from mild reactions such as hives and itching to more lethal outcomes like anaphylaxis, a severe life-threatening immune reaction [2]. The most common allergenic foods include egg, milk, soy, peanut, and seafood [3]. Most food allergens are proteins, but carbohydrate moieties are the relevant allergenic components seen in meat allergy where there is IgE against galactose-alpha-1,3-galactose [4]. In meat allergy, priming is often driven by an external sensitizer like a tick bite. As compared to conventional food allergies, allergic responses to mammalian meat involves a delayed immune response that takes hours [5].

Allergy management broadly involves avoiding food allergens, undergoing allergen- immunotherapy or taking emergency medications during allergic reactions [6]. Comprehensive studies suggest an age bias in food allergies with infants [7,8] having more allergies than adults [9]. Also, there is higher prevalence of allergies in women than men after adolescence [10]. Most allergies to food are primed early during childhood between infancy and age 3 when much of the allergen-specific affinity-matured antibodies are accumulated [11]. This is associated with the infant’s higher intestinal permeability, increased expression of peptide and sugar transporters at birth that declines during fetal life and adulthood [12,13,14]. This may allow an increased entry of allergens to the small intestine compared to adults. The developing immune system of infants may also promote sensitization to the ingested food allergens [15].

Nevertheless, very early exposure to egg and peanut allergen, i.e., between 4 and 11 months can enhance allergenic tolerance and decrease the risk of allergies [16]. Conversely, exposure to allergens at an older age may increase the risk of developing allergies [17]. Discrepancies in the risk of allergies with early or late exposure to the allergen may result from environmental and genetic factors and from the timing of allergen exposure. Early susceptibility to a food allergen could promote a short-term or life-long predisposition to the allergic disease. For unknown reasons, some food allergies, like those to milk or egg allergens, are often outgrown after childhood [18], while those to peanuts and tree nuts often persist for life [19]. This could be linked to genetic factors, the nature of the allergen and the immune response to the allergen [20,21,22,23,24,25]. Some allergens may be good generators of persistent immune memory, induce strong activation of T helper cells and promote/suppress regulatory immune cells [26,27,28]. How these factors influence the persistence of an allergy is still unknown.

In the context of type 2 immunity, B cells can undergo differentiation into Be2 cells that secrete IL-4 and other cytokines [29]. IL-4 from Be2 cells, basophils or other non-T cells is required to promote the differentiation of naïve T cells into T helper (Th) 2 cells [30,31,32]. Through the production of IL-4, Th2 cells eventually promote immunoglobulin class switch to IgE [33]. B cells expressing IgE can rapidly differentiate into plasma cells [34].

Class switch recombination (CSR) is the rearrangement of the genes coding for the constant regions of antibodies while retaining their antigen-binding region. CSR enables antibodies to change their effector functions and serum half-lives. This process is associated with the deletion of the gene segments coding for the antibody classes up-stream of the target class. Therefore, CSR is non-reversible. Human IgM+/IgD+, IgA+ and IgG4+ B cells and murine IgM+/IgD+ and IgG1 B cells can class switch to IgE, respectively, but not vice versa. Class switch from IgM/IgD to IgE is called direct, while class switch from human IgA and IgG4 and from murine IgG1 to IgE is called sequential class switch. There is evidence that most IgE in mice is the product of sequential class switch of IgG1+ progenitor cells [35,36]. CSR to IgE may occur not only in secondary lymphoid tissues but also locally in the mucosa of the nasal cavity, stomach and duodenum [37,38]. The induced antibody composition may dictate the induction and inhibition of an allergy, which may partly explain the complexity of the clinical findings. Below, we will discuss the roles of various antibody composition features, and how the generation of these antibodies is controlled. 

## 2. Low and High-Affinity IgE Play Opposing Roles in Food Allergy

IgE is the least abundant among all antibody isotypes in serum [39]. Even after repeated and long-lasting allergen challenges, it represents only a minor fraction of the total allergen-specific antibodies in serum, suggesting that IgE levels are highly regulated [40]. Its low abundance can be attributed to its comparably short serum half-life of approximately 3 days, and the nature of IgE B cells, which exhibit high rates of apoptosis and little proliferation [41,42,43]. Nevertheless, IgE is crucial for the development of type 1 allergic reactions, such as food allergy. Even small quantities of serum IgE efficiently bind to FcεRI receptors on mast cells and basophils. In contrast to its short half-life in serum, IgE bound on mast cells is retained throughout the life cycle of the cell [44]. The FcεRI receptor binds to the Fc region of the IgE molecule with an affinity of 10^10^ M^−1^ [45]. In consequence, IgE arms mast cells and basophils with an antigen-specific receptor, which, after ingestion of food allergens, meditates cross-linking of the FcεRI receptors, eventually leading to the release of histamine and other mediators of the acute allergic reaction [46]. However, the allergenicity of IgE depends on its affinity [47]. Mast cell-bound IgE antibodies with high affinity for their antigens can be cross-linked by trace amounts of antigen. In consequence, the mast cells are activated and may cause allergic anaphylaxis. In contrast, allergen does not efficiently crosslink low-affinity IgE and was shown to prevent anaphylaxis. In addition, low-affinity IgE can/may prevent anaphylaxis through competing with high-affinity IgE for binding to FcεRI receptors [48,49]. Though both high- and low-affinity stimuli of FcεRI elicit similar receptor phosphorylation, the receptor cluster size, mobility and distribution on mast cells and the down-stream signaling cascade mediated are different, eventually resulting in an altered mast cell response [50]. In accordance with their differential capabilities for mast cell activation, the levels of high-affinity IgE, but not of low-affinity IgE, correlates with allergic symptoms such as eczema, allergic asthma and anaphylaxis [51,52,53].

The physiological role of IgE is poorly understood. It contributes to but is not required for an efficient defense against parasites [54]. Recent data indicate that it is also crucial as a sensor of food quality [55,56]. Natural food consists of a huge number of different substances. In order to minimize uptake of food containing potentially harmful or even toxic molecules, the body has several molecular sensors in the gut that can recognize certain dangerous molecules to provide an early warning to the nervous system, leading to avoidance of this type of food in the future. However, these classical sensors of food quality could recognize only a limited number of harmful substances, leading to nausea and subsequent avoidance behavior. Now, there is evidence that IgE is another sensor of food quality, which potentially can recognize an unlimited number of potentially harmful substances to promote allergen avoidance behavior [55,56,57]. Of note, food quality sensing requires less IgE-mediated mast cell activation than the development of clinically relevant allergic symptoms and seems to be a feature of the early IgE response. It is possible that early, low-affinity IgE is sufficient as a sensor of food quality, while high-affinity IgE formed only later after repeated allergen exposure and appropriate B cell activation leads to allergy. However, this issue needs further investigation [57]. In conclusion, high-affinity, but not low-affinity IgE drives mast cell activation and allergic anaphylaxis [58].

## 3. The Role of Antibody Isotypes, their Subclasses and Antibody Fc Glycosylation in Food Allergy

Antibodies can exhibit highly diverse functions, ranging from highly inflammatory to anti-inflammatory, and from allergenic to anti-allergenic [39,59,60,61,62]. The distinct and partly opposing functions of antibodies are based on the high level of heterogeneity antibodies have with respect to isotype subclass, antigen affinity and Fc *N*-glycosylation pattern. These properties determine their effector functions, i.e., neutralization, opsonization, activation of particular effector cells, complement activation, tissue localization and eventually their pro- or anti-allergenic capacities [59,61,62,63,64,65,66]. The immune reaction to food antigens generates antibodies of various subclasses and affinities, and temporal changes in the relative ratios of allergenic and protective antibodies seem to have a significant impact on the course of allergy development. 

### 3.1. Mechanisms of IgG-Mediated Suppression of Allergy

While IgE is of major importance for allergy development, in the presence of high allergen doses, IgG-mediated anaphylaxis was also observed in murine models [62]. Murine IgG1, IgG2a and IgGG2b have been shown to promote anaphylaxis through activation of the activating Fcγ receptors (FcγR) FcγRI, FcγRIII and FcγRIV [67]. IgG-dependent allergic reactions are mediated through the secretion of platelet-activating factor (PAF) by neutrophils, monocytes, macrophages and basophils [68]. However, this process requires much higher antigen doses than IgE-mediated anaphylaxis [69]. Whether IgG-mediated anaphylaxis is relevant in patients is a matter of debate [70]. In this context, the food quality sensing function of IgE could be relevant. Food sensing is mediated through low-level mast cell activation, which precedes allergic inflammation and promotes a behavior of allergen avoidance. It has been shown that in the absence of IgE or mast cells, allergen uptake is not avoided, eventually leading to gut inflammation mediated by immune effector mechanisms and antibodies other than IgE [56]. Hence, a low-level, subclinical IgE response may help to avoid the uptake of large allergen quantities required for IgG-mediated anaphylaxis.

Though allergen-specific IgG could be potentially harmful, it often seems to be beneficial for allergic patients. Accordingly, increasing levels of allergen-specific IgGs are associated with the natural resolution of food allergies [70]. Likewise, a positive response to allergen-specific immunotherapy is associated with increased allergen-specific serum IgG [71]. Serum IgG4 is elevated in patients who undergo allergen immunotherapy and has been associated with increased clinical tolerance to specific allergens [72]. Depletion of serum IgG4 from peanut-tolerant patients has been shown to promote stronger mast cell degranulation [71]. 

The protective effect of IgG is mediated by multiple mechanisms. IgG can block IgE-mediated allergies via allergen neutralization and FcγRIIb-mediated inhibition via the IgG inhibitory receptor FcγRIIb [73,74,75]. In allergen neutralization, IgG competes with IgE for binding to the allergenic proteins eventually preventing their interaction with IgE. This is an important mechanism by which antibodies provide clinical tolerance to allergic diseases [76]. IgG4 (in humans) and IgG1 (in mice) are clonally related to IgE. Therefore, their antigen-binding sites share the same fine specificity which might be important for efficient competition for allergen binding. In the body fluids, IgG antibodies are present at very higher levels, typically exceeding that of IgE approximately 100 fold or more [35]. The unique blocking properties of IgG4 are associated with their ability to form a Fab arm exchange which allows bispecific antigen recognition thereby interrupting the crosslinking of identical antigens and preventing the formation of immune complexes [77]. Also, IgG4 is unable to activate complement C1q [78]. Accordingly, the administration of blocking murine IgG1 against allergens has also been shown to inhibit IgE-mediated anaphylaxis in mice [75]. 

In addition to its neutralizing activity, IgG can also interact with the FcγRIIb receptor on mast cells and basophils, thereby inhibiting the allergen-IgE-FcεRI activation cascade. Both allergen neutralization and FcγRIIb cross-linking seem to be relevant for IgG-mediated inhibition of IgE-mediated anaphylaxis [75].

### 3.2. Mechanisms of IgA-Mediated Suppression of Allergy

Allergen-specific IgA is also capable of allergen neutralization and is relevant to block IgE-mediated activation of mast cells and basophils [74]. As shown in human samples, a considerable proportion of mucosal IgE is clonally related to IgA [79], indicating that IgA and IgE antibodies share the same antigen binding regions. Mucosal IgA is mostly produced as a dimer that is actively transported to the extracorporeal surface of mucosal epithelial cells [39]. As shown in a murine model of oral immunotherapy, IgA in the mucosal sites binds to allergens and prevents them from penetrating the epithelial barrier and triggering an immune response [74]. Therefore, mucosal IgA can bind to food allergens before they can reach cell-bound IgE and prevents mast cell and basophil degradation in an allergen-specific manner [74]. Since the induction of the most severe and potentially lethal consequence of allergy, systemic anaphylaxis, requires systemic absorption of the ingested allergen [80], the capability of mucosal IgA for allergen neutralization prior to its ingestion, might be of particular relevance for protection from severe allergic reactions. In summary, the protective role of allergen-specific antibodies of IgA and IgG in food allergy and their underlying mechanisms of protection are well documented [73].

## 4. The Impact of Antibody Ig-Fc Glycosylation on Allergy Development

Differential Fc *N*-glycosylation at Asn297 of IgG antibodies modulate their binding to activating and inhibitory Fc receptors and inconsequence their impact on the activation or inhibition of innate effector cells, including mast cells [81]. In inflammatory (auto)immune diseases, IgG antibodies with low levels of galactose and sialic acid have been shown to correlate with disease severity. Consistent with the fact that protein glycosylation is an ancient evolutionary development and that sialylated proteins are more likely to be associated with tolerance, non-galactosylated and non-sialylated forms of IgA antibodies have also been shown to be associated with inflammatory processes [82].

Allergen-specific IgG subclass glycosylation may also play a role in the inhibition of IgE responses via cross-linking with the IgG inhibitory receptor FcγRIIb or, in the presence of high allergen concentrations, in the induction of IgG-mediated allergic reactions via activating FcγRs [83].

In addition, overall (total) IgG Fc glycosylation may play an important role in the control of IgE- and IgG-mediated allergic reactions. An increase in individuals with higher baseline inflammatory immune states (e.g., obesity, unhealthy diet, altered metabolome and microbiome), which is characterized by low levels of galactosylation and sialylation, may be responsible for more frequent shifts to allergic inflammatory phenotypes. The level of Fc galactosylation and sialylation of the overall (total) serum IgG acts as a vast immunological buffer system by regulating the expression of activating and inhibitory FcγRs and can be controlled, for example, by pregnancy and IVIg treatment [59,61,63,84,85,86].

Since IgE is a highly glycosylated antibody isotype, it is very likely that the action of IgE is regulated by its type of glycosylation. In contrast to IgG antibodies, which have one conserved *N*-glycosylation site at Asn 297 in the Fc portion, murine IgE has nine and human IgE has seven potential *N*-glycosylation sites [87]. One site does not appear to be coupled by a glycan. The other sites coupled by glycans are characterized by a conserved pentasaccharide structure of 4 *N*-acethylglucosamines (GlucNAcs) and three mannoses. One of these glycans is of the high mannose type, which is important for IgE binding to the FcƐRI [88]. The other core glycans are of the complex type and can be further modified with a core fucose, a bisecting *N*-acetylglucosamine (GlcNAc), as well as one or two galactose residues, each of which can be further capped by a sialic acid [88,89].

**Figure 1:**
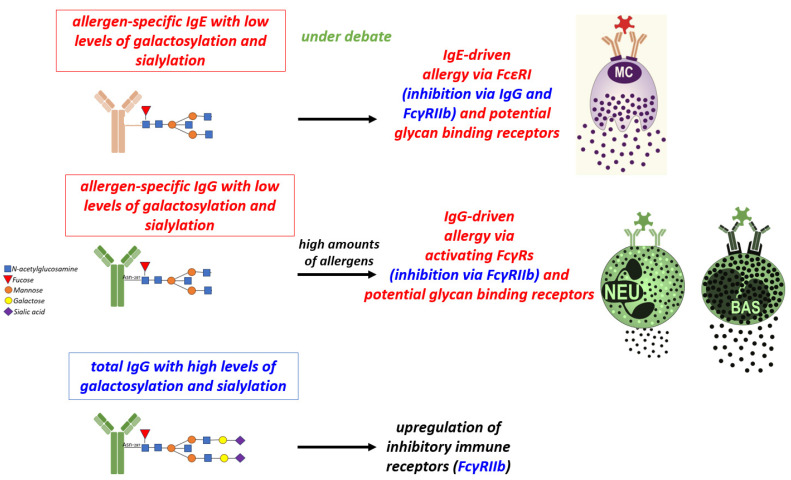
Role of IgE and IgG antibodies and Fc glycosylation in allergy: Allergen-specific IgG can inhibit IgE-mediated allergy via the inhibitory FcγRIIb receptor, but can induce allergy itself via activating FcγRs on neutrophils, basophils and macrophages when allergen amounts are high. First studies suggest that IgG-induced allergy is enhanced by low levels of IgG galactosylation and sialylation. High levels of total IgG galactosylation and sialylation promote upregulation of inhibitory immune receptor FcγRIIb. High-affinity IgE activates mast cells via FcεRI. The function of differentially glycosylated IgE in allergy is still under debate. IgE: Immunoglobulin E, IgG: Immunoglobulin G, MC: mast cells; NEU: neutrophils; BAS: basophils; FcεRI: Fc epsilon RI; FcγRIIb: Fc-gamma RII-b; Fcγ: Fc-gamma.

Instead, a recent study claimed that IgE antibodies with high levels of galactosylation and sialylation are associated with allergic severity [90]. In contrast, our work has shown that non-sialylated IgE antibodies have a greater potential to activate mast cells and basophils [91]. Further work is needed to show how IgE glycosylation evolves over the course of allergy severity or after therapy and how differentially glycosylated IgE antibodies function (Figure 1). Also, the interaction of differentially glycosylated IgE antibodies with FcƐRI(a) and also with soluble or membrane-bound glycan-binding molecules such as IgE-binding protein (galectin-3) and CD33, which may be differentially expressed in different inflammatory conditions, needs to be further investigated [92,93].

## 5. Development of Antibodies in Food Allergy

### 5.1. T Cell Activation

Antibody responses to proteins, such as food allergens, are strictly T-dependent [94]. While B cells recognize three-dimensional epitopes, Th cells are specific for small peptides presented in MHCII molecules. The initial entrance of the allergen leading to specific sensitization may occur through the skin, gastrointestinal tract, airway or damaged epidermal barrier [95,96,97,98]. In allergic individuals, sensitization results in the formation of Th2 and T follicular helper (Tfh) 13 cells and the formation of allergenic IgE [52]. Subsequently, allergenic re-encounter through food ingestion via the gastrointestinal tract leads to IgE-mediated activation of mast cells and basophils, eventually driving acute allergic symptoms.

Food allergens can access a dysfunctional epithelial barrier and trigger the production of alarmins such as Interleukin 33 (IL33), Interleukin 25 (IL25) and Thymic stromal lymphopoietin (TSLP). These alarmins mediate type 1 hypersensitivity [99] by skewing the T cell response towards the Th2 axis and activating mast cells, dendritic cells, innate lymphoid cells and eosinophils [100,101]. B cells may also support the generation of Th2 cells and allergy development through the production of IL-4, as recently shown in a murine model for allergic asthma [30]. 

### 5.2. Production of Unmutated, Low-Affinity IgE

During a response to a protein antigen, such as food allergens, activated B cells can follow multiple differentiation pathways. Initially, the extrafollicular pathway of B cell differentiation yields short-lived plasma cells that produce antibodies of relatively low affinity. B cells following this pathway do not introduce much hypermutations into their antigen-binding regions, nor do they differentiate into memory cells [102,103]. Though extrafollicular B cell differentiation might be mainly important during the initial response, it seems to persist for longer periods at least on low level. In a murine model to food allergy, approximately 5% of IgE clones with a considerable expansion rate (more than 50 copies per clone) did not show signs of hypermutation, even after repeated and long-lasting allergen challenge [35], hence indicating that an extrafollicular response could continue on a low level during established allergy. The extent to which this unmutated, low-affinity IgE may contribute to the inhibition of allergic symptoms has been described above but remains to be further elucidated. At least, low-affinity IgE seems not to correlate with allergic symptoms [51,52]. 

### 5.3. Production of Mutated, High-Affinity IgE

**Figure 2:**
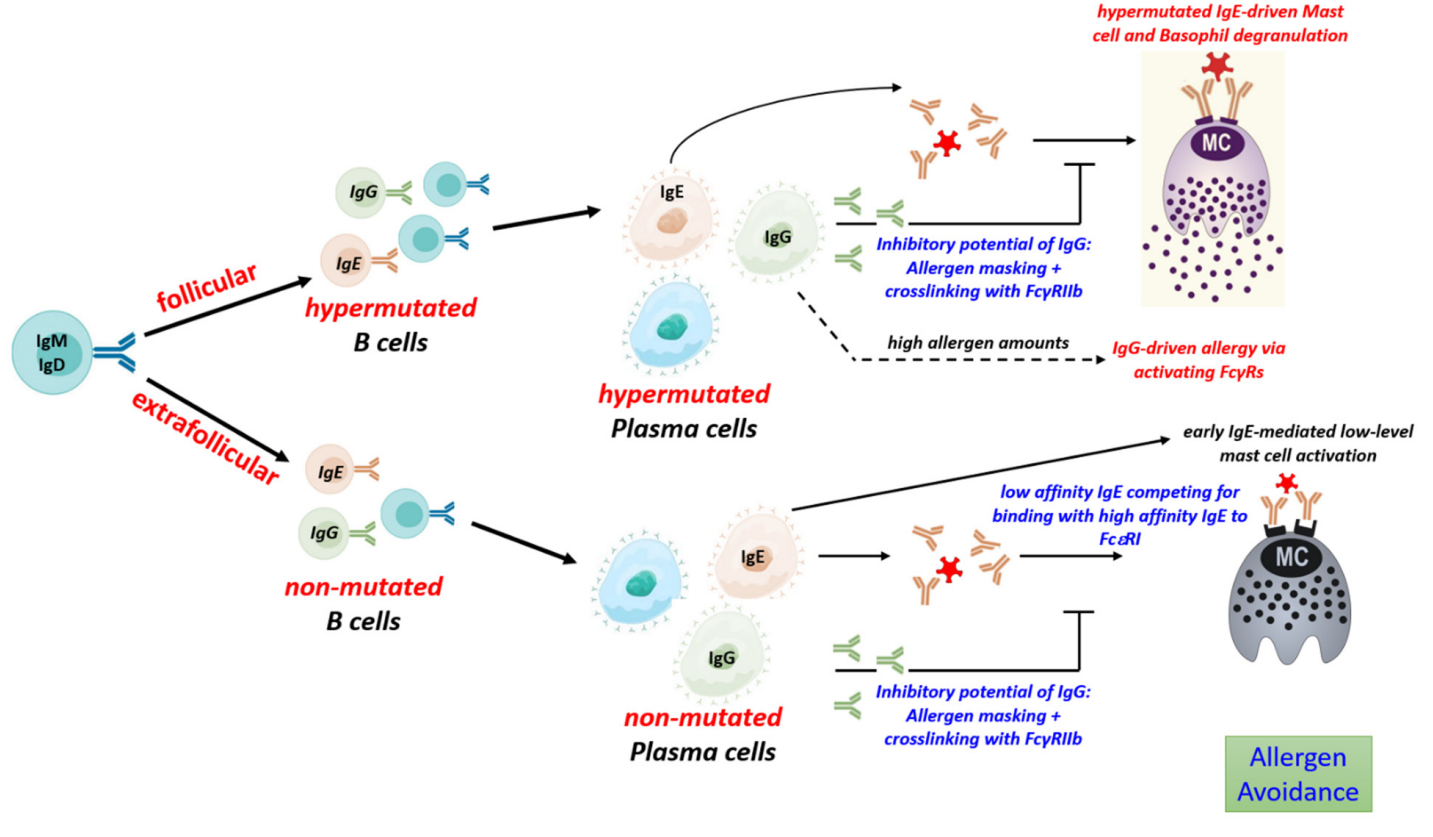
The B cell response to allergens and its impact on mast cell activation. Immune response to food allergens induces follicular and extrafollicular B cell response which yield plasma cells secreting hypermutated (high-affinity) and unmutated (low-affinity) IgE, and IgG with differential impact on the activation of mast cells and disease development. Ig: immunoglobulin; IgE: Immunoglobulin E; IgG: Immunoglobulin G; IgM: Immunoglobulin M; IgD: Immunoglobulin D; FcεRI: Fc epsilon RI; FcγRIIb: Fc-gamma RIIb.

Expressed in a membrane-bound form on the cell surface, antibodies serve as antigen-specific B cell receptor (BCR) which determine the cellular fate at all stages of development [104]. Hypermutated, high-affinity antibodies are the product of the follicular pathway. B cells following the follicular pathway transform primary B cell follicles within secondary lymphoid tissues into germinal centers (GC), where B cells undergo hypermutation, affinity maturation and differentiation into long-lived plasma cells and memory B cells [105,106] (Figure 2). 

GC development requires help from Tfh cells, which provide stimuli such as IL-21 and CD40 essential for induction of hypermutation and positive selection of B cells that have acquired BCR of higher affinity [107,108]. While Th-derived IL-4 is sufficient to induce class switch to IgE and the formation of low-affinity IgE, the generation of high-affinity IgE with anaphylactic properties depends on help from Tfh13 cells which additionally produce high levels of IL-13 and IL-5 together with some IL-21 [52]. Tfh13 cells regulate germinal center responses in type 2 immune reactions and appears to be important for the generation of hypermutated high-affinity IgE and the development of asthma [52]. Genetically modified mice lacking Tfh13 cells show only very low levels of anaphylactic, high-affinity IgE. Tfh13 cells are found in allergic mice and humans with high-affinity IgE to allergens and are further characterized by the expression of the transcription factors BCL6 and GATA3. These cells may represent an interesting target for future therapies of food allergy [109].

A key property of the GC reaction is the generation of memory, mediated by memory B cells and long-lived plasma cells [110]. The pool of long-lived plasma cells secretes antibodies of very high affinity but consist of only a low number of distinct clones. Therefore, they have a limited antigen-binding repertoire [111]. In contrast, memory B cells do not secrete antibodies but provide a backup. Upon antigenic re-stimulation, they can undergo rapid differentiation into antibody-secreting plasma cells. Though the affinities of their antibodies are lower compared to that of long-lived plasma cells, they consist of a higher number of clones that cover a large antigen-binding repertoire [111]. 

Despite the high-affinity IgE is derived from GCs, the existence of IgE+ memory B cells and long-lived plasma cells is a matter of debate. At least the majority of IgE cells seem to be excluded from these memory compartments. Because of their very low frequency and the possibility of confusion with B cells that bind IgE via their low-affinity receptor CD23, IgE-expressing B cells are difficult to detect without doubt. In addition, GCs typically stain brightly for IgG, but only some IgE is detectable in these tissue structures. Thus, the production of IgE appears to be tightly regulated [112]. Different antibody classes exhibit qualitatively distinct signaling properties. In a model using forced IgE BCR signaling has been shown to induce apoptosis, independent from antigenic stimulation [43]. Accordingly, in the same study, primary IgE+ cells showed a higher rate of apoptosis than IgG1+ cells. It is noteworthy that another study confirmed the finding that IgE expression mediates a tonic, antigen-independent signal. This study could not confirm that IgE directly promotes B cell apoptosis; instead, independent of antigen binding, it was found to support terminal differentiation into plasma cells, which involves multiple parts of the IgE BCRs as well as Syk, CD19, BLNK, Btk and IRF4 [41]. 

Another study showed that though IgE is formed by reactivation of IgG memory cells, signaling of the membrane IgE BCR, but not of the murine IgG1 BCR, is required to yield high IgE levels [113]. IgE BCR expression was still found on plasma cells, which is different to IgG, which is not expressed any more on the surface of long-lived plasma cells, and IgE signaling on plasma cells was found to be relevant for the production of serum IgE.

Together, IgE BCR expression mediates a tonic signal even in the absence of external stimulation by antigen, if that promotes apoptosis or terminal differentiation might be dependent on the model, and under physiological conditions, on additional factors such as the availability of an anti-apoptotic environment. Some IgE+ B cells seem to survive and signal via their membrane-bound IgE receptor which is a crucial regulator of IgE production.

Comparison of IgE+ and IgG1+ murine B cells by whole-genome CRISPR screening showed that IgE+ B cells have distinct properties [114]. Different form IgG+ cells, IgE+ B cells and IgE+ plasma cells showed chronic calcium signaling eventually resulting in BCL2L11-dependent apoptosis. Moreover, there is evidence that after repeated antigenic stimulation in mice, high-affinity IgE-secreting plasma cells are generated through reactivation and further class switch recombination of IgG1 memory B cells [36]. Together, these findings indicate that the majority of IgE is formed by short-lived plasma cells generated from IgG+ memory B cells.

Nevertheless, next-generation sequencing of the IgG1 and IgE repertoires in a murine model of food allergy indicates that IgE+ cells may follow individual fates. While most IgE clones showed little clonal expansion, a small proportion of highly hypermutated IgE clones exhibited massive clonal expansion, comparable to that of the most expanded IgG1 clones [35]. These data are in accordance with the view that most IgE+ cells show little proliferation and exhibit a short lifetime. But a few hypermutated, GC-derived IgE+ B cells seem to undergo positive selection and proliferation. If these expanded IgE clones that escape from early apoptosis can survive on the long run to enter the memory compartment remains to be elucidated. 

In favor of such an idea, there is some evidence for the existence of IgE+ memory B cells and long-lived plasma cells in mice and humans [115,116,117]. 

### 5.4. Regulation of IgG to IgE Ratios

**Figure 3:**
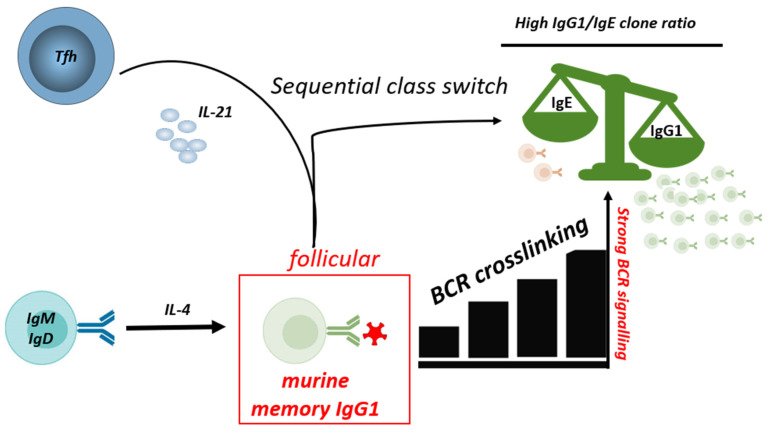
BCR signaling strength and IL-21 affect IgE/IgG1 ratios. Strong BCR signaling of mouse IgG1 B cells and IL-21 from Tfh cells constraints sequential class switch to IgE, thereby reducing the IgE/IgG1 ratio. IgE: Immunoglobulin E; IgG1: Immunoglobulin G1; IgM: Immunoglobulin M; IgD: Immunoglobulin D; BCR: B cell receptor; IL-4: Interleukin 4; IL-21: Interleukin 21.

Most of the class-switched IgE+ cells are derived from IgG+ B cells that underwent further (sequential) class switch to IgE in a follicular B cell response [33,35,118]. The mechanisms controlling the relative IgE to IgG production during the allergen-specific immune response are only partly understood so far. Studies from our laboratory investigating the antibody response to hen’s egg in a murine food allergy model indicate that IgE-to-IgG ratios are controlled on the level of single B cell clones [35]. Most individual clones containing both IgE and IgG1, showed a several-fold excess of IgG1 compared to IgE, i.e., a high IgG1-to-IgE ratio. However, fewer, but still a considerable proportion of clones showed a massive excess of IgE, with ratios above 5-fold more IgE than IgG1. Evidence was provided that the differential IgG1 to IgE ratios are due to individual BCR signaling strength which had two consequences. First, strong BCR signaling inhibited sequential class switch from IgG1 to IgE. Second, BCR crosslinking could optimize help from T follicular helper cells producing IL-21, a cytokine that was found to favor IgG1 over IgE production. Hence, on a clonal level, IgG1-to-IgE ratios seem to be strongly affected by the individual antibody affinities [35] (Figure 3). There is increasing evidence that nutrition and metabolic factors can have a strong impact on B cell activation [119]; however, their role on class switch, and the relative ratios of allergenic IgE and anti-allergenic IgG remains to be elucidated. 

## 6. Development of Differentially Glycosylated Antibodies

IgG Fc glycosylation (galactosylation and sialylation) are regulated by two glycosyltransferases, β1,4-galactosyltransferase 1 (B4galt1) and α2,6-sialyltransferase 1 (St6gal1), in antibody-producing B cells [120]. There is evidence that the expression of these enzymes and hence IgG Fc glycosylation is controlled by Tfh cell-derived cytokines [121]. Within the GC, IL-6/IL-23-dependent IL-17A+ Tfh17 cells induce a low IgG Fc sialylation program in B cells. How these mechanisms affect the glycosylation of antibodies in the context of allergies is not known. 

Early IgE antibodies from extrafollicular plasma cell responses show low hypermutation rates with correspondingly low affinity [35]. These plasma cells may generate IgE antibodies with high levels of galactosylation and sialylation as shown for early extrafollicular IgG antibodies after immunization [121,122]. IgG antibodies derived from the germinal center show higher mutation rates and lower levels of galactosylation and sialylation [121]. Depending on the co-stimulation inducing the germinal center response, the derived plasma cells produce IgG antibodies with distinct levels of galactosylation and sialylation, but all lower than the initial extrafollicular level of galactosylation and sialylation [121]. In allergy, germinal center-derived plasma cells may produce different IgE/IgG ratios as well as IgE (and IgG) antibodies with reduced levels of galactosylation and sialylation, which may also depend on the co-stimulation that induce the germinal center response. Tfh13 cells have recently been linked to IgE antibodies in asthma [52,123]. It remains to be investigated which Tfh cell subsets induce which IgE glycosylation profile. Whether inflammatory Tfh13 cells or other Tfh cell subsets, such like Tfh17 cells, which are important for the induction of inflammatory glycosylated IgG antibodies [121,124], can influence the development of inflammatory IgE glycosylation patterns during the germinal center reaction has to be investigated. Different allergen immunotherapies may also induce different Tfh and GC B cell responses and IgE/IgG(4) ratios, as well as IgE (and IgG) antibodies with different glycosylation levels, depending on the type of adjuvant [83]. Accordingly, anti-cytokine therapies that affect the germinal center response may affect the IgE/IgG ratio and/or the IgE (and IgG) glycosylation pattern.

## 7. The Impact of Distinct Antibody Types in Type 1 Allergic Reactions to Aero-Allergens

Cross-reactivity between aero-allergens and food allergens is considered to be relevant for the development of food allergies in patients previously sensitized to aero-allergens. E.g., initial exposure to respiratory allergens from plant pollens can promote secondary allergy to different food allergens. This complicates the diagnosis of some food allergies originating from pollen cross-reactivity [125]. Other inhalant-allergens (also called aero-allergens) include house dust mites, animal dander, pollen, dust, etc. [126]. Similarly to what is seen in food allergies, the antibody response in aero-allergies includes IgE, IgA and IgG antibodies. Though some earlier studies linked the severity of allergic airway inflammation to antigen-specific IgG, suggesting that they contribute to the development of allergy to inhalant allergens, other studies have associated IgG4 with improved tolerance to aero-allergens [127]. More recently, mechanistic studies provided convincing evidence that aero-allergen-specific IgG can efficiently suppress allergic reactions. Monoclonal IgG against cat allergen feld1 has been shown to reduce allergic symptoms in mice and patients [128]. Another study suggested that mouse IgG can reduce airway hypersensitivity through interaction with FcγRIIb on dendritic cells [129]. Accordingly, the efficacy of allergen immunotherapy widely used for the treatment of various aero-allergies such as allergic rhinoconjunctivitis or allergic asthma has been linked to the generation of anti-allergenic IgA and IgG [73]. Together, these findings indicate that IgA and IgG antibodies are protective against both food allergies and aero-allergies.

## 8. Conclusions

The contribution of the B cell responses and the induced antibody compositions to the development or protection from food allergy is complex. IL-4+ Th2 cells are sufficient to drive the production of low-affinity, potentially protective IgE, but the generation of high-allergenic high-affinity IgE within GCs requires additional help from Tfh13 cells. Allergen-specific IgG and IgA antibodies limit IgE-mediated allergic symptoms in patients. Recent results imply that Ig Fc *N*-glycosylation imprinted during the GC reaction may also have a considerable impact on their pro/anti-allergenic properties.

## Data Availability

Not applicable.
